# Enhanced HIV-1 Control After Antibody Therapy is Associated with Autologous Antibodies and Reservoir Clearance in the RIO trial

**DOI:** 10.1101/2025.11.03.25339415

**Published:** 2025-11-06

**Authors:** Marcilio J. Fumagalli, Anna Kaczynska, Marius Allombert, Ágata Lopes-Ribeiro, Mariângela de Oliveira Silva, Brianna J. Hernandez, Christy Lavine, Sebastian M. Espinosa, Helen Brown, Hanna Box, Emanuela Falaschetti, Louise-Rae Cherrill, Tamara Elliott, Ming Lee, Julie Fox, Sarah Pett, Amanda Clarke, Alison Uriel, Ole Sogaard, Rebecca Sutherland, Marta Boffito, Sabine Kinloch-Loes, Chloe Orkin, Simon Collins, Panagiota Zacharopoulou, Nicola Robison, Cintia Bittar, Thiago Oliveira, Anna Gazumyan, Mila Jankovic, Michael S. Seaman, John Frater, Marina Caskey, Sarah Fidler, Michel C. Nussenzweig

**Affiliations:** 1 Laboratory of Molecular Immunology, The Rockefeller University, New York, NY USA.; 2 Humoral Immunology Unit, Institute Pasteur, Université Paris Cité, Paris, France.; 3 Laboratory of Basic and Applied Virology, Microbiology department, Institute of Biological Sciences, Federal University of Minas Gerais, Belo Horizonte, MG, Brazil; 4 Laboratory of Antigen Targeting to Dendritic Cells, Parasitology Department, University of São Paulo, São Paulo, SP, Brazil.; 5 Center for Virology and Vaccine Research, Beth Israel Deaconess Medical Center, Harvard Medical School, Boston, MA, USA.; 6 Nuffield Department of Medicine, University of Oxford, Oxford, UK and the Oxford NIHR Biomedical Research Centre, Oxford, UK.; 7 School of Public Health, Imperial Clinical Trials Unit, Imperial College London, London, UK.; 8 NIHR Imperial Biomedical Research Centre, Imperial College London, London, UK.; 9 Department of Infectious Disease, Imperial College London, London, UK.; 10 Guy’s and St Thomas Hospital NHS Foundation Trust, London, UK.; 11 University College London, London, UK.; 12 Central and Northwest London NHS Foundation Trust, London, UK.; 13 University Hospitals Sussex NHS Foundation Trust, Brighton, UK.; 14 Brighton & Sussex Medical School, Brighton, UK.; 15 Manchester University NHS Foundation Trust, Manchester, UK.; 16 Department of Clinical Medicine, Aarhus University Hospital, Aarhus, Denmark.; 17 Western General Hospital, NHS Lothian Trust, Edinburgh, UK; 18 Chelsea and Westminster Hospital, London, UK; 19 Royal Free London NHS Foundation Trust, London, UK.; 20 Queen Mary University of London, London, UK.; 21 Barts Health NHS Trust, London, UK.; 22 HIV i-Base, London, UK; 23 Department of Genetics, Ribeirao Preto Medical School, University of Sao Paulo, Ribeirão Preto, SP, Brazil.; 24 NIHR Oxford Biomedical Research Centre, University of Oxford, Oxford, UK.; 25 Howard Hughes Medical Institute, The Rockefeller University, New York, NY, USA

## Abstract

RIO is an ongoing double blind randomized placebo controlled human trial in which participants that started antiretroviral therapy (ART) during primary or early-stage infection underwent treatment interruption and were randomly assigned to a group receiving one or two doses of two long acting broadly neutralizing antibodies 3BNC117-LS and 10–1074-LS (Arm A), or saline (Arm B). The two arms differed significantly in time to viral rebound, ART restart and number of individuals that remained off therapy after 96 weeks (p=0.001)^1^. Here we report on the relationship between viremic control, the latent HIV-1 proviral reservoir, rebound viruses and their sensitivity to the infused and autologous antibodies. Pre-infusion reservoir measurements showed low levels of intact proviral HIV-1 DNA in circulating CD4+ T cells in both arms. The rebounding viruses in participants that received the antibodies, showed significant selection for resistance to 10–1074-LS but not to 3BNC117-LS. Notably, there was a significant correlation between initial reservoir sensitivity to autologous antibodies and to 10–1074 and time to rebound. Finally, comparison of pre-infusion and pre-rebound HIV-1 proviral reservoir in participants that received antibodies showed that the reservoir of intact proviruses decayed faster than earlier reports with a half-life of 0.65 years.

## Introduction

Upon entry into CD4+ T cells the HIV-1 RNA genome is reverse transcribed and preferentially integrated into transcriptionally active sites in the host genome^2^. High level transcription of replication-competent proviruses leads to cell death and, if untreated, almost invariably results in lethal immunodeficiency^3^. Anti-retroviral therapy (ART) prevents the spread of infection and immunodeficiency but fails to eliminate a reservoir of latent proviruses found primarily in long-lived, resting memory CD4+ T cells^4–7^. These reservoir cells express viral RNA at levels below the threshold required to either induce cytopathic effects or elicit robust immune-mediated clearance, yet they can retain the capacity to reinitiate systemic infection upon ART interruption^8,9^.

The HIV-1 reservoir contains both intact and defective proviruses and has been characterized by several different methods including viral outgrowth assays, digital droplet polymerase chain reaction (ddPCR), and whole genome amplification and sequencing (Q4PCR)^10–13^. Whereas the size of the defective reservoir is relatively stable over time on ART, the intact reservoir shows early rapid decay which slows to a half-life of approximately 4.5 years, with possible further prolongation after 7 years on therapy^14–17^. Intact proviruses remaining in the reservoir after long-term therapy tend to reside in areas of the genome that are less transcriptionally active^18–20^. Although the mechanism of selection against the reservoir viruses integrated into transcriptionally active parts of the genome is not precisely understood; selection is thought to be mediated by a combination of HIV-1 cytopathic effects and host immune responses.

Passively administered antibodies can enhance host immune responses and induce simian HIV control by a CD8^+^ T cell-dependent mechanism^21–23^. In addition, several small studies in humans have reported that broadly neutralizing antibody (bNAb) therapy is associated with accelerated clearance of infected cells, delayed viral rebound and a higher-than-expected rate of prolonged control^24–29^. However, the mechanism of post-bNAb control in humans remains poorly understood. Control has been associated with increased HIV-1 specific CD8^+^ T cell responses in some but not all studies^24–27^ and tissue culture experiments suggest that autologous antibodies may also put pressure on the reservoir^30^.

**Extended data Figure 1.**
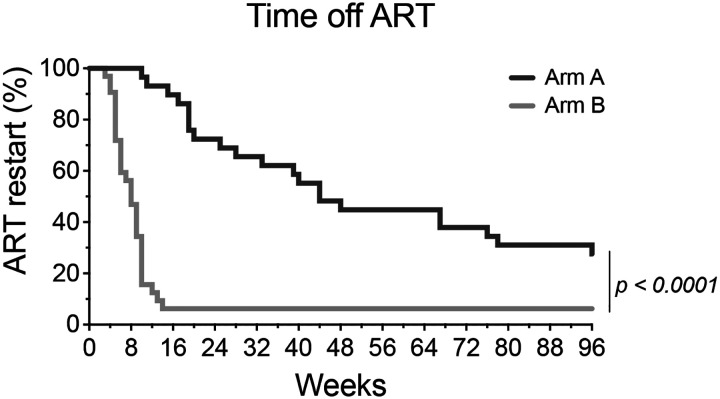
Kaplan Meyer curve shows time to ART restart (up to 96 weeks) among participants in Arms A (n=29) and B (n=32). The curves were compared using the Gehan–Breslow–Wilcoxon test (Chi-square = 34.43, *P* < 0.0001).

The RIO trial is an ongoing 1:1 randomized placebo-controlled trial that has recruited 68 participants^1^. Eligible volunteers received ART during primary or early infection and remained on suppressive therapy for a minimum of 1 year and a median of 5 years before study entry. Participants in Arm A received one or two doses of 2 long acting bNAbs, 3BNC117-LS and 10–1074-LS, at least 20 weeks apart (median time 27 weeks), while those in Arm B received saline. All participants underwent treatment interruption two days after the first dose. Remarkably, 24% (7/29) of the participants that had received bNAbs and remained in follow up according to protocol, controlled viremia (plasma HIV-1 RNA < 1,000 copies/mL) beyond 96 weeks after ART interruption compared to 6% (2/32) in the control group (p=0.001^1^ and Extended data Fig. 1). Here, we report the viral reservoir characteristics, dynamics and antibody sensitivity in the RIO trial.

## Results

### Baseline reservoir.

To examine the HIV-1 proviral reservoir in individuals enrolled in the RIO trial, we obtained pre-intervention peripheral blood mononuclear cells. Reservoir size was estimated by ddPCR and Q4PCR^10,11^. ddPCR documents the presence of 2 conserved regions in the provirus, the packaging signal and envelope^10^. It is more sensitive, less expensive and scalable than Q4PCR^11^. However, it does not allow sequence confirmation and therefore some of the proviruses that are assigned as intact are in fact defective, a problem that can increase with time because the intact reservoir decays faster than the defective^31^. Q4PCR provides sequence information and direct confirmation of whether a provirus is intact or defective, but it is far less sensitive than ddPCR because it requires amplification of the 9 kilobase proviral genome^32^.

**Extended data Figure 2.**
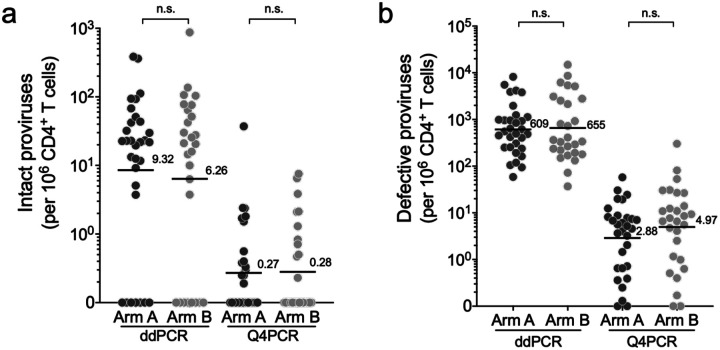
Baseline HIV reservoir in circulating CD4+ T cells measured by ddPCR and Q4PCR. Graphs show the numbers of (**a**) intact and (**b**) defective proviral genomes per million CD4+ T cells. Each dot represents the value for one participant. Arm A participants black and Arm B grey. Sample size: ddPCR – Arm A, *n* = 31; Arm B, *n* = 27; Q4PCR – Arm A, *n* = 30; Arm B, *n* = 27. The black line and numbers indicate the geometric mean. Group comparisons were performed using the Mann–Whitney test, with statistical significance defined as *P* < 0.05.

To examine the pre-intervention reservoir in the RIO participants we performed both assays. Individuals in the 2 arms (ddPCR: Arm A n=31, Arm B n=27, Q4PCR: Arm A n = 30, Arm B n= 28) showed similar numbers of intact and defective proviruses in ddPCR and Q4PCR assays (Extended data Fig. 2a and b, and Supplementary Table 1). The geometric mean number of intact proviruses per million CD4+ T cells was 9.32 and 6.26 by ddPCR (p=0.5), and 0.27 and 0.29 by Q4PCR (p=0.5), for Arms A and B, respectively (Extended data Fig. 2a). The relatively small size of the reservoir in this cohort is consistent with early therapy^33–35^.

**Figure 1.**
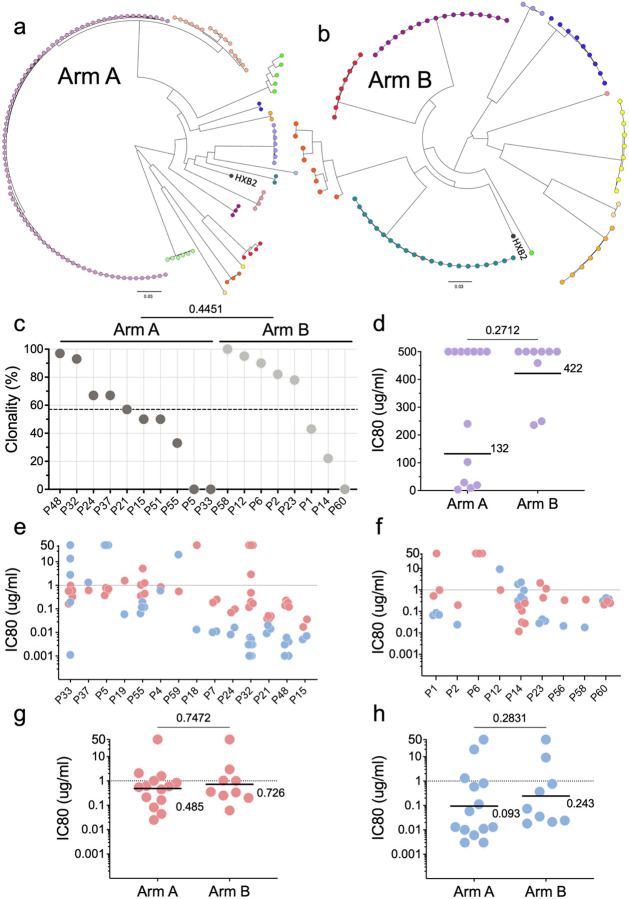
Baseline reservoir characteristics. **a,b**, Circular phylogenetic trees using the generalized time-reversible (GTR) model depict clonal distribution of intact proviral nucleotide sequences based on *env* (**a**) Arm A and (**b**) Arm B. Colors represent different participants. **c**, Intact reservoir clonality for the indicated participants, calculated based on amino acid sequences of *env* for individuals with ≥3 intact proviral sequences. Dashed line indicates combined average clonality of both Arms. **d**, Baseline reservoir sensitivity to pre-infusion autologous IgG antibodies, expressed as IC80; each dot represents the geometric mean sensitivity of proviruses obtained from one individual. Line and numbers show overall geometric mean for all participants in Arms A (n=13) and B (n=9). **e,f**, Baseline reservoir neutralization sensitivities to 3BNC117 (red) and 10–1074 (blue), expressed as IC80. Dotted lines indicate 1 mg/ml. **e**, Individual pseudovirus IC80s for the indicated participants in Arm A. **f**, Individual pseudovirus IC80s for the indicated participants in Arm B. **g,h**, Each dot represents the geometric mean IC80s for all pseudoviruses from an individual participant against (**g**) 3BNC117 and (**h**) 10–1074. Arm A n=14 and Arm B n=9. Group comparisons in **c,d** and **g,h** were performed using the Mann–Whitney test, with statistical significance defined as *P* < 0.05.

We were able to recover 141 and 91 intact proviral reservoir sequences from baseline samples from 15 and 11 individuals in Arms A and B, respectively (Fig. 1a and b, Supplementary Table 2). As expected, each participant harbored a separate non-overlapping group of intact HIV-1 proviral sequences (Fig. 1a and b). Despite early therapy and the small reservoir sizes, intact proviruses showed a median level of clonality of 57% when considering all individuals from whom we were able to obtain three or more intact proviral sequences^12,36–38^ (Fig. 1c and Supplementary Table 2).

Sixty-five representative pseudotype viruses from the reservoir of 14 and 9 individuals in Arms A and B, respectively, were tested for neutralization sensitivity to autologous baseline IgG, 3BNC117, and 10–1074 in TZM-bl assays^39^ (Fig. 1d–h and Supplementary Tables 2, 3 and 6). Consistent with early therapeutic intervention, only 8 of these 23 individuals showed autologous antibody neutralizing activity with an IC80s <250 ug/ml (or ≅1:100 dilution of serum) against their reservoir proviruses (Fig. 1d, and Supplementary Tables 3 and 6).

Reservoir sensitivity to each of the two bNAbs, ranged from an IC80s of <0.001 to >50 ug/ml for 10–1074 and 0.012 to >50 ug/ml for 3BNC117, with no significant differences between the two study arms (Fig. 1 e–h). The geometric mean IC80s were 0.485 ug/ml and 0.726 ug/ml for 3BNC117 and 0.093 and 0.243 for 10–1074 for Arms A and B, respectively (Fig. 1g and h and Supplementary Table 6). All individuals in the bNAb arm showed reservoir proviral sensitivity to at least 1 of the 2 bNAbs (Supplementary Table 3).

### Viral reservoir and rebound dynamics upon treatment interruption.

All RIO trial participants underwent treatment interruption^1^. Treatment was restarted if the viral load was >100,000 HIV-1 copies/ml for 2 consecutive weeks, or > 1,000 HIV-1 copies/ml for 6 weeks or CD4 T cell count <350 cells/ml, or clinical symptoms or participant preference^1^. Participants in the control arm (Arm B) experienced viral rebound after an average of 4.6 weeks with only 2 out of 32 (6%) remaining undetectable throughout 96 weeks of observation^1^. The mean time to ART re-start was 8.4 weeks in the control arm. The mean time to ART restart in the bNAb arm was 47.4 weeks, with 8 out of 29 remaining off ART at 96 weeks (p<0.0001, Extended data Fig. 1). Notably 13 of the 29 individuals re-started ART before meeting virological ART restart criteria with 50% of the remaining group exhibiting control for at least 96 weeks (8/16)^1^.

**Extended data Figure 3.**
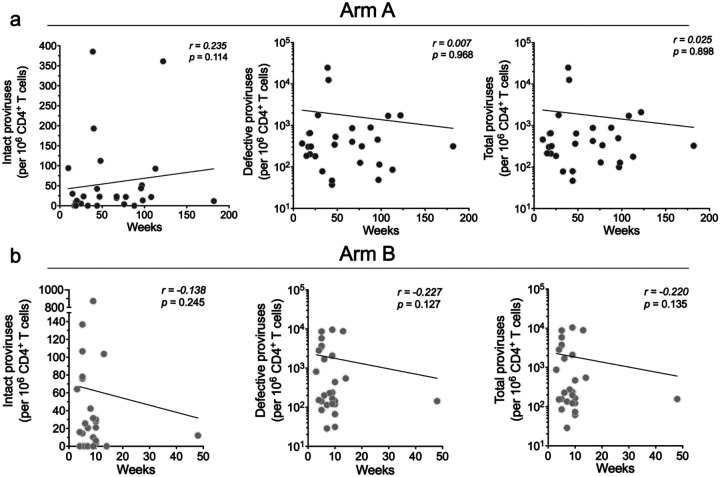
Correlation between baseline intact, defective and total reservoir size and time to ART restart in weeks measured by ddPCR. **a**, Arm A. **b**, Arm B. Each dot represents one individual. n = 28 for Arm A and n = 27 for Arm B. Reservoir size was measured by digital droplet PCR. Correlation coefficients were calculated using Spearman’s *r* with 95% confidence intervals. Nonlinear curve fitting was performed with *x* as a linear function, and *y* as a linear or logarithmic function for intact and defective genomes, respectively.

**Extended data Figure 4.**
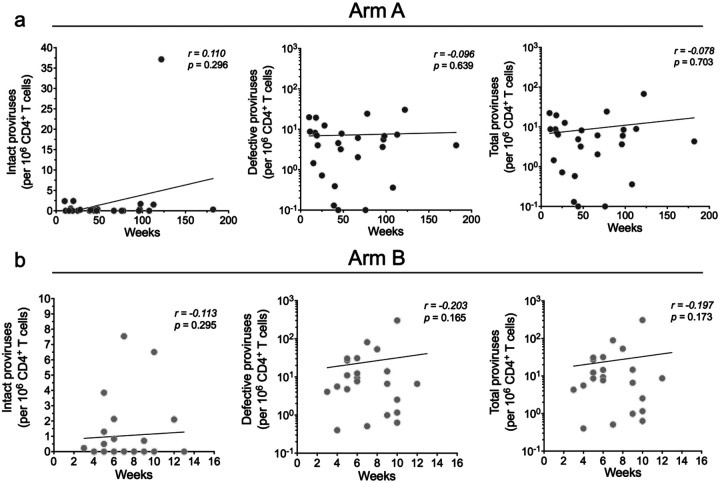
Correlation between baseline intact, defective, and total proviral reservoir size and time to ART restart weeks measured by Q4PCR. (**a**) Arm A and (**b**) Arm B. Each dot represents one individual. Reservoir size was measured by Q4PCR. Correlation coefficients were calculated using Spearman’s *r* with 95% confidence intervals. Nonlinear curve fitting was performed with *x* as a linear function, and *y* as a linear or logarithmic function for intact and defective genomes, respectively.

**Extended data Figure 5.**
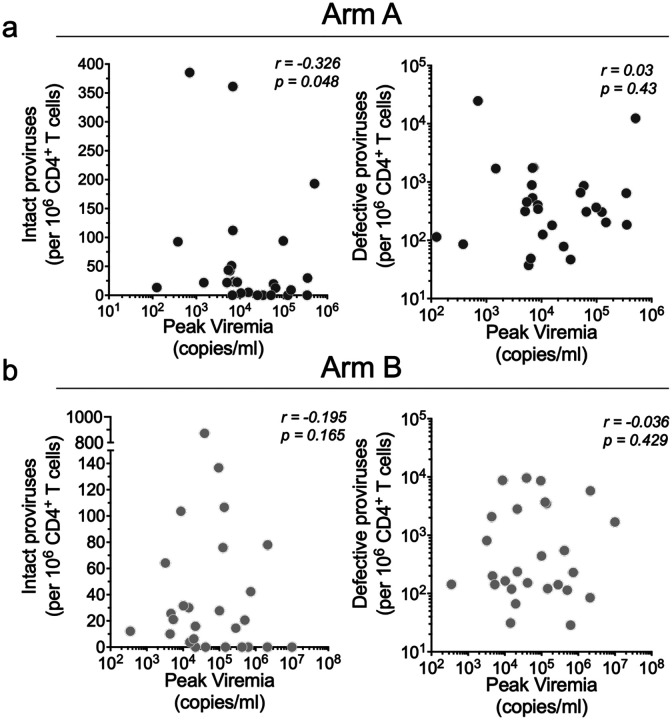
Relationship between reservoir size measured at baseline by ddPCR and peak viremia. (**a**) Arm A and (**b**) Arm B. Each dot represents one individual. Y axis shows number of intact or defective proviruses and X axis viremia at the time of peak rebound. Correlation coefficients were calculated using Spearman’s *r* with 95% confidence intervals.

There was no significant correlation between the size of the intact proviral reservoir and time to ART restart in the placebo or bNAb arms of the study, as measured by either ddPCR or Q4PCR (Extended data Fig. 3 and 4). In addition, there was no correlation between the reservoir size and peak viremia prior to ART restart in either group (Extended data Fig. 5).

### Rebound viremia.

**Figure 2.**
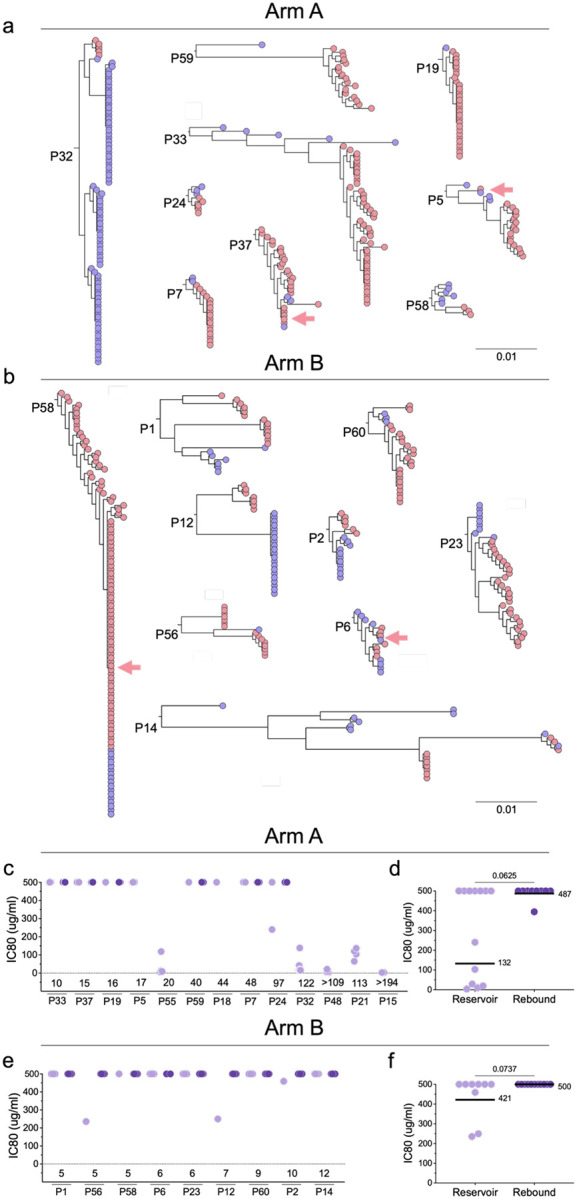
Relationship between intact proviral reservoir and rebound *env* nucleotide sequences from participants in both study arms. **a,b**, Phylogenetic trees were constructed using the generalized time-reversible (GTR) model for participants in Arm A and Arm B. Intact reservoir proviruses are shown in purple and rebound in red. Red arrows indicate sequences shared between reservoir and rebound. **c-f**, Neutralization of reservoir and rebound pseudoviruses by autologous antibodies in participants from Arm A and Arm B. (**c, e**) Individual pseudovirus IC80s. (**d, f**) Each dot represents the geometric mean IC80 for all pseudoviruses from one participant. Arm A reservoir n=13 and rebound n=9. Arm B reservoir n=9 and rebound n=11. Line and numbers indicate geometric mean for all participants. Group comparisons in (**d**) and (**f**) were performed using the Mann–Whitney test, with statistical significance defined as *P* < 0.05.

**Figure 3.**
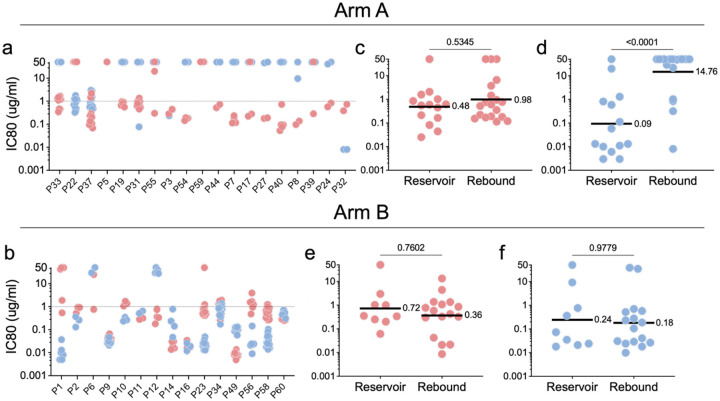
Pseudovirus neutralization by 3BNC117 (red) and 10–1074 (blue) for participants expressed as IC80. **a,b**, Each dot represents an individual rebound pseudovirus for Arm A and Arm B. **c-f**, Each dot represents the geometric mean IC80 for reservoir- and rebound pseudoviruses in one participant Arm A: (**c**) 3BNC117 and (**d**) 10–1074 (reservoir n=14, rebound n=19), and Arm B: (**e**) 3BNC117 and (**f**) 10–1074 (reservoir n=9, rebound n=16). Line and numbers indicate geometric mean for all participants. Group comparisons in (**c,d**) and (**e,f**) were performed using the Mann–Whitney test, with statistical significance defined as *P* < 0.05.

We were able to obtain 323 and 370 rebound viral *env* sequences by single genome amplification, from 19 and 16 individuals who experienced viral rebound in Arms A and B, respectively (Supplementary Table 4). To examine the nature of rebound viremia and its relationship to the proviral reservoir, we compared reservoir and rebound *env* sequences from 18 individuals from whom we were able to obtain both intact reservoir and rebound sequences (Fig. 2a and b). Representative pseudotype viruses were produced from rebound viral sequences from participants in Arms A (96) and B (119), respectively and were tested for sensitivity to the 2 bNAbs and to autologous antibodies obtained at the time of entry (Fig. 2c – f, Fig. 3a–f and Supplementary Tables 4 and 5).

Rebound virus sequences rarely overlap with the dominant latent intact proviral clones found in the circulating reservoir^40,41^. This phenomenon remains poorly understood but has been attributed in part to suppression of reservoir viruses by autologous antibodies^30,42^. Among 33 intact latent proviral reservoir clones (91 sequences) obtained from 9 control arm participants, we found only 2 that overlapped with rebound sequences (Fig. 2b). There was no overlap between the reservoir and rebound sequences in the remaining 7 control participants, only 3 of whom had measurable levels of autologous neutralizing antibodies to the reservoir (Fig. 2e and Supplementary Tables 3 and 5). Thus, autologous antibody neutralizing activity cannot entirely account for the disparity between intact proviruses found in the reservoir and the dominant viruses found at the time of rebound in this study of individuals receiving early ART therapy. Nevertheless, rebound viruses in the 3 individuals from the control group that had measurable autologous neutralizing antibodies to the reservoir were resistant to their own antibodies (Fig 2d–f). Thus, when autologous antibodies are present, at baseline (during ART) rebound viruses are enriched in variants that are autologous antibody resistant.

**Extended data Figure 6.**
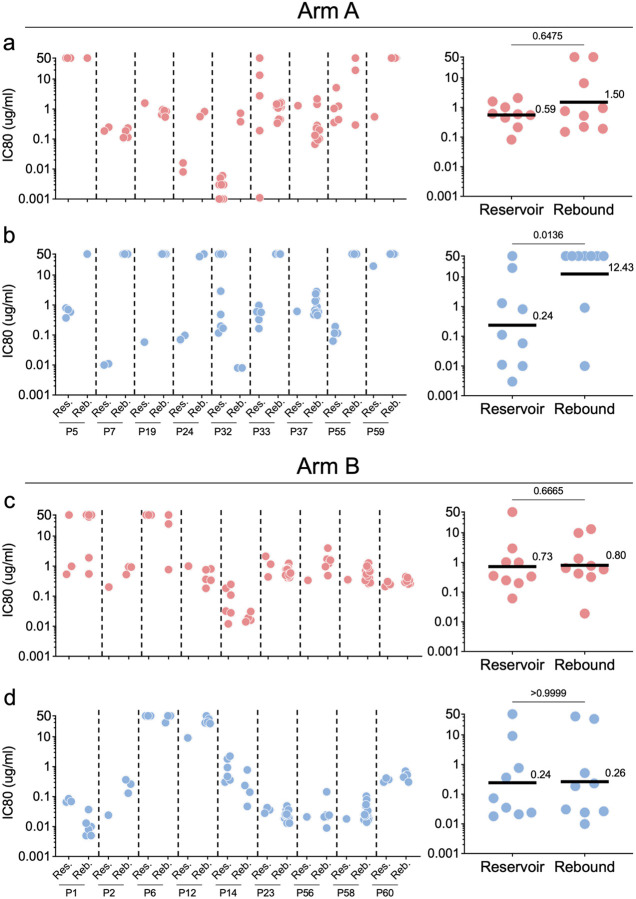
Per-participant matched reservoir- and rebound-derived pseudovirus neutralization sensitivities to 3BNC117 and 10–1074 in both study arms, expressed as IC80 values. **a-d,** Each dot represents a reservoir- or rebound-provirus from each participant in (**a, b**) Arm A and (**c, d**) Arm B. N = 27 participants for each Arm. Members of expanded clones are represented only once irrespective of the size of the clone. Geometric mean IC80 values of all reservoir- and rebound-derived clones per participant are indicated alongside each graph. Group comparisons were performed using the Mann–Whitney test, with statistical significance defined as *P* < 0.05. Black lines indicate the geometric mean.

Pseudotype viruses obtained from rebound were also tested for sensitivity to the 2 bNAbs (Fig. 3a–f). Consistent with the absence of selective pressure in the control arm, the geometric mean bNAb sensitivity of all reservoir and rebound pseudotype viruses obtained from Arm B participants was not significantly different for either antibody (3BNC117 p=0.76, 10–1074 p=0.98, Fig. 3b, e and f and Extended data Fig. 6c and d).

In contrast, selection for bNAb resistance was evident among rebound viruses obtained from Arm A participants (Fig. 3a, c and d). The geometric mean neutralizing IC80s for 10–1074 against reservoir and rebound pseudoviruses was 0.09 and 14.7 mg/ml, respectively with the majority of rebound viruses being completely resistant (IC80 > 50 mcg/mL) (p<0.0001 Fig. 3d and Extended data Fig. 6b). In contrast, there was a much smaller difference in the geometric mean IC80s between reservoir and rebound for 3BNC117 but that did not reach statistical significance (p=0.53, Fig. 3c and Extended data Fig. 6a). Only 3 of 19 participants that received bNAbs showed rebound viruses that were uniformly resistant to 3BNC117 (Fig. 3a and Supplementary Table 5). In conclusion, 10–1074 escape variants were far more likely to emerge than 3BNC117 resistant variants during dual bNAb therapy, as reported in earlier studies^25,28,43^.

### Viral Control and Reservoir Antibody Sensitivity.

**Figure 4.**
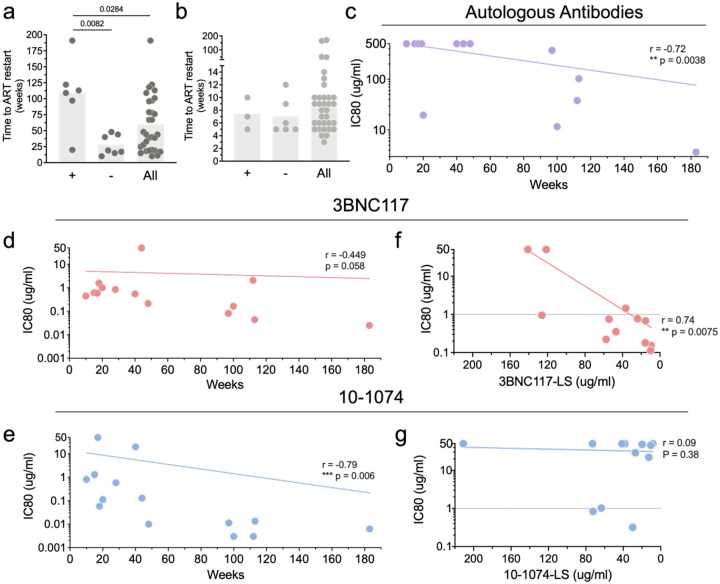
Viral suppression and reservoir sensitivity to autologous antibodies, 3BNC117 and 10–1074. **a,b**, Time to ART restart for individuals that did (+) or did not (−) display reservoir sensitivity to autologous antibodies or all participants. (**a**) Arm A and (**b**) Arm B. Each dot represents the time to rebound for an individual participant. The bars represent the geometric mean for each group. Mann–Whitney group comparisons, with *P* < 0.05 considered significant. **c-e**, Correlation between time to ART restart and baseline reservoir sensitivity to (**c**) autologous antibodies, (**d**) 3BNC117, and (**e**) 10–1074. Each dot represents the geometric mean IC80 (Y axis) and time to rebound in weeks (X axis) for one participant. **f,g**, Correlation between IC80 for (**f**) 3BNC117 or (**g**) 10–1074 and the concentration at the time of rebound. Each dot represents the geometric mean IC80 (Y axis) and bNAb concentration at the time of rebound in micrograms per milliliter (X axis) for one participant. Nonlinear curve fitting was performed with *x* as a linear function of time or bNAb concentration, and *y* as a logarithmic function of IC80 values. Correlation coefficients were calculated using Spearman’s *r* with 95% confidence intervals.

To determine whether reservoir sensitivity to either autologous antibodies or bNAbs was associated with control of viremia, we compared baseline autologous neutralizing titers and bNAb IC80s to reservoir viruses and time of ART restart (Fig. 4 a–g). The mean time to ART restart among the three individuals in the control group that exhibited autologous antibody neutralizing activity against reservoir viruses was 7.3 compared to 8.5 weeks to all participants (Fig. 4b). Thus, we see no measurable effect of the autologous antibodies in the absence of bNAb therapy. In contrast, the mean time to ART restart in the six bNAb recipients that showed baseline autologous neutralizing activity was 108 weeks compared to 27.5 to the 13 that did not (p=0.008, Fig. 4a). Consistent with this observation, there was a significant correlation between pre-infusion autologous neutralizing antibody titers to reservoir viruses and time to rebound for individuals in the bNAb arm (n=13, r=−72 p=0.004, Fig. 4c). Time to rebound was also correlated with initial reservoir proviral sensitivity to 10–1074 (r=−0.79, p=0.006) but there was only a trend for 3BNC117 (r=−0.4, p=0.06, Fig. 4d and e). Notably, there was an inverse correlation between 3BNC117 concentration at the time of rebound and the IC80 of the rebounding virus to the bNAb (Fig 4f). In contrast, this relationship was not observed for 10–1074, which displayed a broad range of concentrations at rebound (Fig. 4g). These results are consistent with the idea that individuals that rebound do so when they develop resistance to 10–1074 and have subtherapeutic concentrations of 3BNC117 in circulation. Although the number of reservoir viruses analyzed was a small fraction of the total present in any given individual, the data indicates that the probability of escape from 10–1074 was directly related to the sensitivity of the intact proviruses in the initial reservoir (Fig. 4e).

**Extended data Figure 7.**
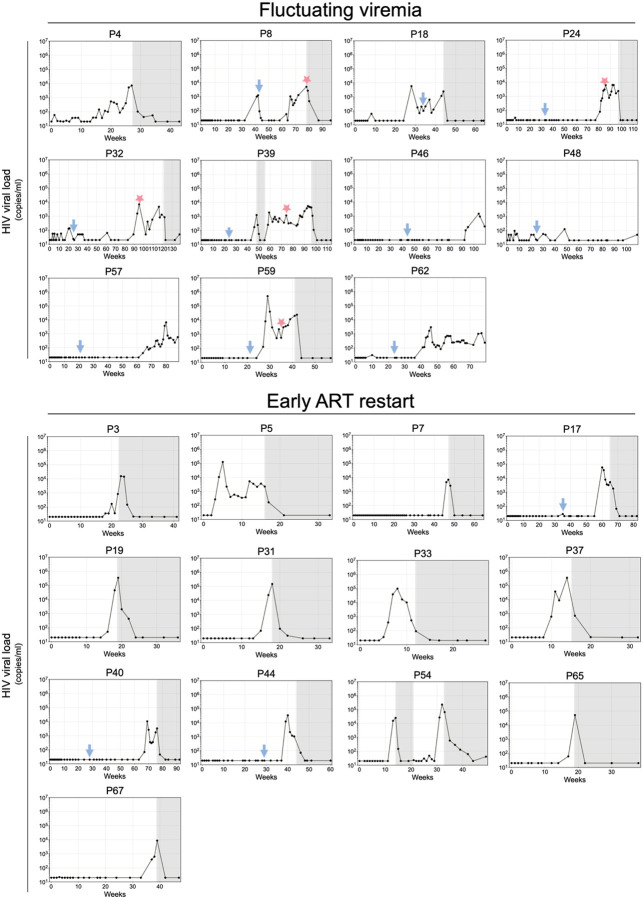
Longitudinal viral loads in participants with fluctuating viremia (more than 16 weeks without sustained HIV-1 RNA > 1,000 copies/ml); early ART restart (2 consecutive VLs ≥10^5^ or 6 consecutive VLs ≥10^3^). X axis time in weeks and y axis HIV-1 copies per milliliter. Blue arrows indicate a second bNAb infusion, and red stars indicate rebound viral sequencing and pseudovirus generation for neutralization assays. Gray shading indicates periods on ART.

**Extended data Figure 8.**
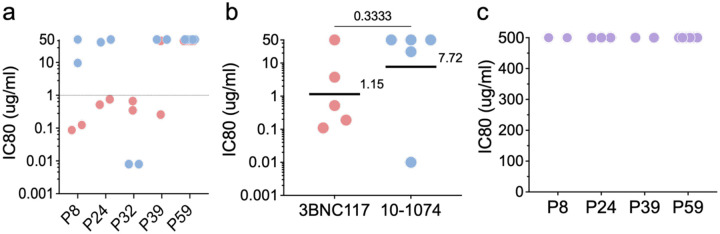
Neutralization of pseudovirus obtained during sustained viremia. **a-c**, Graphs show iC80s for (**a**) individual pseudoviruses (**b**) geometric mean per participant (**c**) individuals pseudoviruses against pre-infusion autologous antibodies. 3BNC117 (red), 10–1074 (blue), and pre-infusion autologous IgG (purple). Lines and numbers indicate the geometric mean across the participants. Group comparisons in (**b**) were performed using the Mann–Whitney test, with statistical significance defined as *P* < 0.05

Among the 30 Arm A participants that had remained in follow up according to protocol, 14 went back on ART before meeting virological restart criteria (2 consecutive VLs ≥10^5^ HIV-1 copies/ml or 6 consecutive VLs ≥10^3^). Of the 16 remaining, 11 (69%) maintained fluctuating viremia and did not meet ART re-start criteria for 16 to >58 weeks (Extended data Fig. 7). In contrast, only 2 of the Arm B control showed prolonged fluctuating viremia^1^. Only 5 participants viruses were obtained from time points associated with fluctuating viremia, and all were resistant to pre-infusion autologous antibodies when present. In addition, 4 of these individuals showed viruses that were resistant to 10–1074, but 3 retained sensitivity to 3BNC117 (Extended data Fig. 8). Thus, antibodies may play a role in maintaining long-term fluctuating persistent viremia, but monotherapy with 3BNC117 was insufficient to maintain fluctuating viremia in other studies, suggesting the existence of additional mediators of enhanced control^11,28,29^.

### Reservoir dynamics.

**Figure 5.**
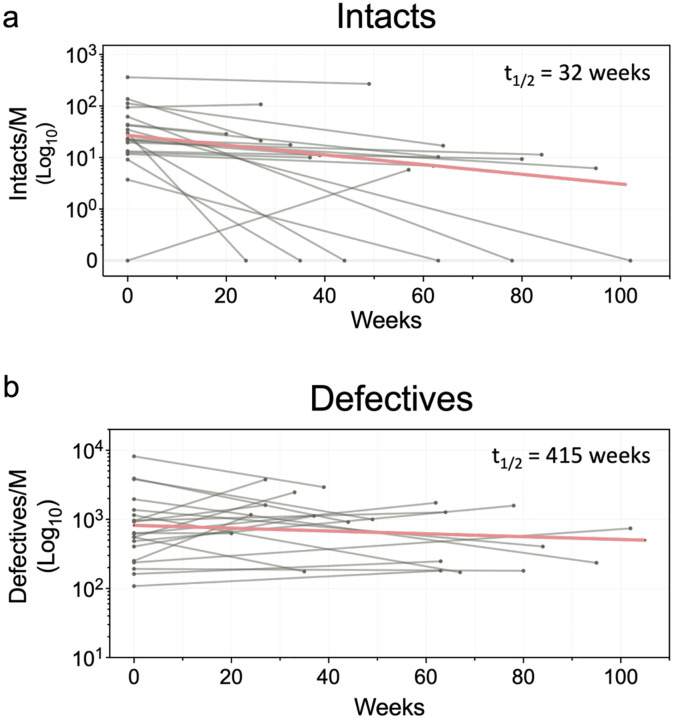
Half-life decay rates of the HIV reservoir for participants enrolled in Arm A. **a,b,** Graphs show number of intact or defective proviruses as measured by digital droplet PCR (Y axis) at baseline and time after first infusion in weeks (X axis). n=20 participants. Half-life decay for both intact and defective proviral compartments was calculated using a log-linear regression model.

To determine how bNAb therapy impacts the size of the proviral reservoir we compared the intact and defective proviral reservoirs at baseline with pre-rebound samples collected at a mean of 52 weeks after the initial infusion (n=20, Fig. 5 and Supplementary Table 1). We used ddPCR for these measurements because the relatively small reservoir size limited the number of samples from which we were able to obtain paired Q4PCR measurements (Supplementary Table 1). Although, there was a great deal of variation between individuals over time, the intact and defective reservoirs had half-lives of 32 and 415 weeks, respectively (n=20, Fig.5a and b). Thus, antibody therapy in the RIO trial was associated with an average 0.65-year intact and 8.6-year defective reservoir half-lives. Although the defective reservoir half-life is compatible with prior measurements, the intact half-life is shorter than prior published reservoir measurements and irrespective of the method of analysis^44^.

## Discussion

RIO is an ongoing double-blind, randomized placebo-controlled human trial in which participants that started ART during primary or early infection were randomly assigned to a group that received 3BNC117-LS and 10–1074-LS, Arm A, and a control group, Arm B, and subsequently discontinued ART. Despite similar pre-infusion reservoir measurements, the groups differed significantly in time to rebound^1^, and in the nature of the rebounding viruses. After 96 weeks 23% and 6% of the individuals in the active and control arms, respectively remained off ART. Prolonged control was associated with pre-existing autologous neutralizing antibodies and sensitivity to 10–1074. Paired pre-infusion and pre-rebound intact reservoir measurements in participants that received bNAbs showed that their intact reservoir decayed with a half-life of 0.65 years.

As expected, based on their relatively early start on therapy, participants in RIO had small HIV-1 proviral reservoirs^33–35^. Nevertheless, most of the intact reservoir in participants was clonal and did not contribute to rebound^40^. Why reservoir proviruses are rarely found among rebound viruses is not entirely understood, but there is an inverse correlation between the size of an expanded clone and the probability that the sequence will be found among rebound viruses^12^. The difference has been attributed to selection for proviral integration into silent sites in the genome^45^, and to autologous antibody mediated suppression^30,42^. However, the disparity between reservoir and rebound viruses was evident in the control arm individuals that did not have measurable autologous antibodies or receive bNAbs. Thus, the discrepancy between reservoir proviruses in circulation and rebound viruses cannot entirely be explained by autologous antibody suppression of viremia in this cohort of early treated individuals.

HIV-1 infection is associated with the development of antibodies that neutralize the autologous virus and continually select for viruses that express resistant variants during the course of the infection^46,47^. Moreover, autologous antibodies can sieve the outgrowth of reservoir viruses *in vitro*^30,42^. But although autologous antibodies alter the viral swarm by selection of resistant variants, they fail to control viremia, suggesting that antibodies are ineffective against HIV-1. This idea was further supported by the observation that passively administered first generation bNAbs had little, if any, measurable effect on infection^48–50^.

Subsequent experiments in mice and macaques showed that administration of more potent bNAbs suppresses viremia and elicits CD8+ T cell responses that lead to prolonged control of infection^21–23,51^. In addition, small interventional studies in which people living with HIV-1 received second generation bNAbs demonstrated that antibodies can be both safe and effective in reducing viremia in the absence of ART^43,52–55^. Notably, a fraction of individuals receiving bNAbs during treatment interruption appear to control the infection for an unexpectedly long time^24–26,28,29,56,57^.

Enhanced CD8+ T cell responses were associated with bNAb therapy in some but not all reported clinical studies and therefore other mechanisms are thought to contribute to enhanced control^24,55,58^. The data obtained from RIO indicates that individuals that have a pre-existing autologous neutralizing antibodies are more likely to control for longer periods of time. In theory, autologous antibodies could function as an adjunct to the 2 bNAbs making it more difficult for the virus to escape from combined antibody pressure^59^. The autologous antibody responses could contribute to control in a few different ways including but not limited to: 1. direct neutralization; 2. viral and infected cell clearance; 3. enhancing antigen presentation to elicit cellular immune responses.

A significant fraction of the participants in the bNAb arm of the RIO trial maintained fluctuating viremia for 16 to >58 weeks in the face of complete or partial bNAb resistance (11 of 29 or 38% of Arm A). Although it did not reach significance, enhanced post-bNAb control has also been recently reported in the TITAN trial and in a single arm study of 10 individuals that received bNAbs, a TLR agonist and a vaccine^24,25,28^. The precise mechanistic explanation for these observations is not well defined, but the role of residual antibody cannot be dismissed. We speculate that enhanced post-bNAb control is at least in part due to blunting of the initial surge in viremia by innate, cellular and humoral immunity, thereby preserving the host immune system and its ability to partially control viremia.

Antibody monotherapy therapy like small molecule therapy results in selection for resistant variants. Combination therapy durably suppresses viremia because the probability of developing escape mutations at a combination of non-overlapping different sites is less likely. Consistent with this idea, rebound typically occurred in the RIO trial under functional monotherapy. Rebound viruses were most frequently resistant to 10–1074 and pre-existing autologous antibodies. In contrast, selection against 3BNC117 was far more limited with no significant difference between initial reservoir and rebound sensitivity to the antibody, suggesting that the antibody was subtherapeutic at rebound. The median estimated 3BNC117-LS concentration at rebound was 52.4 μg/mL which is similar to the median 3BNC117 IC80 for these participants at the time of rebound (41.8 ug/ml, Fig. 4f), and higher than the estimates in previous small clinical trials^25,28^.

**Extended data Figure 9.**
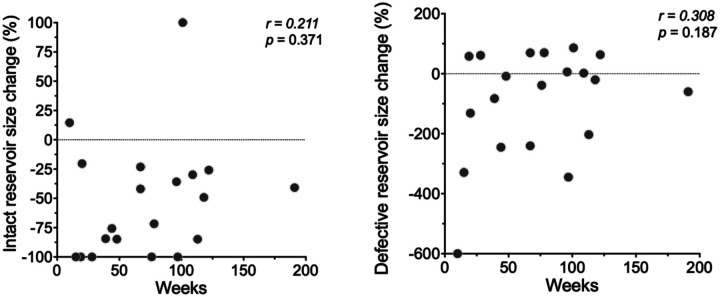
Graphs show absence of correlation between change in reservoir and time to rebound. X ais time in weeks Y axis 5 change in reservoir. Each dot is one participant. Dotted lines show 0% level change relative to pre-infusion baseline. Correlation coefficients were calculated using Spearman’s *r* with 95% confidence intervals.

RIO trial participants who received bNAbs demonstrated accelerated intact reservoir decay, 0.65 years compared to the 4–7 years reported in nearly all studies to date. Accelerated reservoir decay was also a feature of a small study in which participants received multiple doses of a short acting native version of 3BNC117 and 10–1074^25^, but not in other small studies^24,26^. The difference may be due to inherent variability and the small numbers of participants assayed, who were treated during chronic infection as opposed to early therapy participants in the RIO trial, differences in the antibodies used and their half-lives, and finally significant variability in the nature of the reservoir between individuals^11,24,26^. Why some participants in this trial showed only small or no change in reservoir size remains to be determined but may be due to heterogenous and/or variable levels of proviral transcription among CD4+ T cells and even among members of a clone of CD4+ T cells^60–62^. Moreover, the observed changes in reservoir size cannot account for and do not correlate with prolonged antibody-mediated suppression (Extended data Fig. 9). Far greater changes in reservoir would be required to alter the time to rebound in most individuals.

In conclusion, the data establishes that a significant fraction of people living with HIV-1 who receive bNAb therapy can partially control viremia for prolonged periods of time. Correlates of prolonged control of HIV-1 after bNAb therapy include pre-existing autologous antibodies and pre-existing or enhanced CD8+ T cell responses^26^, suggesting that vaccination to elicit or enhance pre-existing host immunity may further increase the number of individuals exhibiting post-bNAb control.

### Limitations of this Study.

Although 68 individuals were recruited into the RIO study, we were unable to obtain sequence information on the HIV-1 reservoir in 33 of the participants, thereby limiting the number of participant samples available for analysis. The analysis was limited by small reservoir size, sample availability and non-B clade viruses. Moreover, sampling was limited to peripheral blood and may not entirely reflect tissue reservoirs. This problem was further accentuated when considering the relationship between autologous antibodies and virologic outcomes because early ART interferes with the development of autologous neutralizing antibody responses. In addition, the timing of sample acquisition was pre-determined and did not always correspond to timepoints of interest limiting analysis of rebound viremia.

## Methods

### Study participants

The RIO trial is a randomized, placebo-controlled, double-blinded phase II study^63^. This study enrolled individuals who initiated ART during primary or early HIV infection (defined as nadir CD4 count > 500 cells/ml) and remained on suppressive ART for at least 1 year. A total of 68 participants enrolled and were randomly assigned in a 1:1 ratio into two arms as part of a two-stage clinical design. Blood samples were collected at baseline and at multiple time points following 3BNC117-LS and 10–1074-LS or placebo infusions. Samples were processed briefly after collection, with serum and plasma stored at −80°C. Peripheral blood mononuclear cells (PBMCs) were isolated by density gradient centrifugation. The PBMC number was determined either manually or using an automated cell counter (Vi-Cell XR; Beckman Coulter), and cells were cryopreserved in liquid nitrogen in fetal bovine serum supplemented with 10% DMSO.

### Intact proviral DNA analysis (IPDA)

The frequency of CD4+ T cells harboring intact, 5’-deleted, and 3’-deleted proviruses was determined using a ddPCR assay modified from the Intact Proviral DNA Assay (IPDA)^10^. Briefly, CD4+ T cells were enriched from PBMCs using a negative immunomagnetic selection kit (Miltenyi Biotec). Genomic DNA was extracted using the QIAamp DNA Mini Kit (Qiagen) according to the manufacturer’s instructions. DNA concentrations were quantified using the Qubit 3.0 Fluorometer with the Qubit dsDNA BR Assay Kit (Thermo Fisher). Samples were from the pre-infusion time point and obtained from an aviremic time from a minimum of six weeks before rebound, when HIV-1 RNA was < 20 copies/ml.

For ddPCR, two sets of primers and probes specific to the HIV Gag and Env regions, along with two fragments of the housekeeping gene RPP30, were utilized in separate reactions^64^. HIV quantification was performed using 750 ng of genomic DNA per sample with the following primers and probes for the HIV Gag region: Forward (5’-GACTAGCGGAGGCTAGAAGGAGAGA-3’), Reverse (5’-CTAATTCTCCCCCGCTTAATAYTGACG-3’), and Probe (5’−6FAM-ATGGGTGCGAGA-IABkFQ-3’). For the HIV Env region: Forward (5’-AGTGGTGCAGAGAGAAAAAAGAGC-3’), Reverse (5’-GTCTGGCCTGTACCGTCAGC-3’), and Probes (5’-VIC-CCTTGGGTTCTTGGGA-MGB-3’ and an unlabeled hypermutated probe 5’-CCTTAGGTTCTTAGGAGC-MGB-3’). An alternative primer/probe set targeting the PS region was used as a backup: Forward (5’-CAGGACTCGGCTTGCTGAAG-3’), Reverse (5’-GCACCCATCTCTCTCCTTCTAGC-3’), and Probe (5’−6FAM-TTTTGGCGTACTCACCAGT-IABkFQ-3’)^10^. To measure input cell numbers and correct for DNA shearing, 7.5 ng of DNA was used with RPP30 primers and probes (RPP30–1: Forward: 5′-GATTTGGACCTGCGAGCG-3′, Reverse: 5′-GCGGCTGTCTCCACAAGT-3′, Probe: 5′−6FAM-TTCTGACCTGAAGGCTCTGCGC-IABkFQ-3′; RPP30–2: Forward: 5′-GTGTGAGTCAATCACTAGACAGAA-3′, Reverse: 5′-AAACTGCAACAACATCATAGAGC-3′, Probe: 5′-HEX-AGAGAGCAACTTCTTCAAGGGCCC-IABkFQ-3′). Four technical replicates were performed per sample. Positive and negative controls were included in each reaction.

The ddPCR assays were conducted using the Bio-Rad QX200 AutoDG system with the ddPCR Supermix for Probes (no dUTPs) (Bio-Rad). Thermal cycling conditions included an initial denaturation at 95°C for 10 minutes, followed by 45 cycles of 94°C for 30 seconds and 59°C for 1 minute, with a 2°C/second ramp rate. A final extension was performed at 98°C for 10 minutes, followed by a hold at 12°C. The results were adjusted for DNA shearing using the ratio of double-positive RPP30 partitions and normalized to 10^6^ CD4+ T cells.

### Quadruplex 4-probes real-time quantitative PCR (Q4PCR)

The Q4PCR assay was conducted to characterize the composition of the reservoir, as previously described^11^. Briefly, total CD4+ T cells were enriched from cryopreserved PBMCs using a negative immunomagnetic selection kit (Miltenyi Biotec). Genomic DNA was extracted from CD4+ T cells using the Gentra Puregene Cell Kit (Qiagen), and DNA concentration was measured using the Qubit dsDNA BR Assay Kit (Thermo Fisher Scientific).

A total range of 1 to 10 × 10^6^ CD4 T cells were screened per participant. An initial outer PCR (NFL1) was performed on genomic DNA at a single-copy dilution using the outer primers BLOuterF (5′-AAATCTCTAGCAGTGGCGCCCGAACAG-3′) and BLOuterR (5′-TGAGGGATCTCTAGTTACCAGAGTC-3′)^65^. A 1-μl aliquot of the undiluted NFL1 PCR product was then subjected to a Q4PCR reaction, utilizing four primer–probe sets targeting conserved regions of the HIV-1 genome. PS forward, 5′-TCTCTCGACGCAGGACTC-3′; reverse, 5′-TCTAGCCTCCGCTAGTCAAA-3′; probe, 5′-/Cy5/TTTGGCGTA/TAO/CTCACCAGTCGCC-3′/IAbRQSp; env forward, 5′-AGTGGTGCAGAGAGAAAAAAGAGC-3′; reverse, 5′-GTCTGGCCTGTACCGTCAGC-3′; probe, 5′-/VIC/CCTTGGGTTCTTGGGA-3′/MGB; gag forward, 5′-ATGTTTTCAGCATTATCAGAAGGA-3′; reverse, 5′- TGCTTGATGTCCCCCCACT-3′; probe, 5′-/6-FAM/CCACCCCAC/ZEN/AAGATTTAAACACCATGCTAA-3′/ IABkFQ; and pol forward, 5′-GCACTTTAAATTTTCCCATTAGTCCTA-3′; reverse, 5′-CAAATTTCTACTAATGCTTTTATTTTTTC-3′; probe, 5′-/NED/AAGCCAGGAATGGATGGCC-3′/ MGB. Quantitative PCR (qPCR) was performed under the following thermal cycling conditions: an initial denaturation at 94 °C for 10 minutes, followed by 40 cycles of 94 °C for 15 seconds and 60 °C for 60 seconds. qPCR assays were conducted in a 384-well plate format using the Applied Biosystem QuantStudio 6 or 7 Flex real-time PCR system. Data analysis was performed using ThermoFisher Design and Analysis Software 2.4.3. Samples that exhibited reactivity with two or more of the four probes were selected for a nested PCR (NFL2).

The NFL2 reaction was performed on undiluted 1-μl aliquots of the NFL1 PCR product. Each reaction was conducted in a 20-μl volume using Platinum Taq high-fidelity polymerase (Thermo Fisher Scientific) and the primers 3LTRi (5′- TCAAGGCAAGCTTTATTGAGGCTTAA-3′) and U5–638F (5′- GCGCCCGAACAGGGACYTGAAARCGAAAG-3′)^19^. The thermocycler conditions for NFL2 were the same as those used for the NFL1 PCR. Library preparation and sequencing were conducted as previously described^11^.

### HIV-1 sequence assembly and annotation

HIV-1 genome reconstruction was performed using an in-house pipeline, Defective and Intact HIV Genome Assembler (DIHIVA), designed for assembling raw sequencing reads into annotated HIV genomes^25^. The pipeline includes rigorous quality control steps to enhance accuracy and reliability. First, quality control checks were conducted to remove PCR-amplified reads and correct sequencing errors using clumpify.sh from the BBtools package v38.72 (http://sourceforge.net/projects/bbmap). Next, adapter sequences and low-quality bases were trimmed, and potential contaminant reads were removed using the Trim Galore package v0.6.4 (https://github.com/FelixKrueger/TrimGalore). HIV-1 sequences were assembled using SPAdes v3.13.0, and the longest assembled contig was aligned to the HXB2 HIV-1 reference genome via BLAST^66^. Sequences exhibiting double peaks—regions indicating the presence of two or more viral variants within a sample (defined by a consensus identity cut-off of <70% for any residue)— or samples with an insufficient number of sequencing reads (≤500 reads) were excluded from downstream analyses.

Sequences that did not meet the double-peak criteria (consensus identity for any residue <70%) were further classified as either intact or defective proviruses. Only intact HIV-1 env sequences were considered for subsequent analyses.

### Single-genome amplification of plasma rebound virus env genes

Sequencing of HIV-1 plasma rebound *env* genes was performed as previously described^67^. In brief, HIV-1 RNA was extracted from viremic plasma samples using the MinElute Virus Spin kit (Qiagen) according to manufactures recommendation. First-strand cDNA synthesis was carried out using SuperScript III reverse transcriptase (Invitrogen) and an antisense primer, envB3out (5′-TTGCTACTTGTGATTGCTCCATGT-3′) for subtype B or OFM19 (5′–GCACTCAAGGCAAGCTTTATTGAGGCTTA-3′) for subtype C. To ensure single-genome amplification, cDNA was endpoint diluted according to Poisson distribution, achieving <30% of wells yielding a PCR product. The envelope gene was then PCR-amplified using subtype B primers envB3out and envB5out (5′-TAGGCATCTCCTATGGCAGGAAGAAG-3′) or subtype C primers OFM19 and Vif1 (5′-GGTTTATTACAGGGACAGCAGAG-3′). A second round of PCR was performed using 1 μl of the first-round PCR product as a template, with subtype B primers envB3in (5′-GTCTCGAGATACTGCTCCCACCC-3′) and envB5in (5′-TAGGCATCTCCTATGGCAGGAAGAAG-3′), or subtype C primers ENV A (5′-GGCTTAGGCATCTCCTATGGCAGGAAGAA-3′) and ENV N (5′-CTGCCAATCAGGGAAGTAGCCTTGTGT-3′). PCR amplifications were conducted using High Fidelity Platinum Taq (Invitrogen) under the following conditions: an initial denaturation at 94°C for 2 minutes, followed by 35 cycles of 94°C for 15 seconds, 55°C for 30 seconds, and 68°C for 4 minutes, with a final extension at 68°C for 10 minutes. PCR products of the expected size were subjected to library preparation and sequencing using the Illumina MiSeq platform.

### Sequence and phylogenetic analysis

Nucleotide alignments of intact env sequences were performed using Muscle v5.1 with PPP algorithm^68^. Sequences containing premature stop codons, truncations, or frameshift mutations were excluded from further analyses. Maximum likelihood phylogenetic trees were generated from these alignments using FastTree 2.1.11 with the GTR model and 1,000 bootstrap replicates^69^.

### Pseudotyped-virus production

Selected single-genome sequences from CD4+ T cell reservoir or plasma rebound samples were used as templates to produce pseudoviruses from a CMV promoter as described^70^. The cytomegalovirus (CMV) promoter was amplified by PCR from pcDNA 3.1 (Life Technologies) using the primers CMVenv (5′-AGTAATCAATTACGGGGTCATTAGTTCAT-3′) and CMVenv1A (5′-CATAGGAGATGCCTAAGCCGGTGGAGCTCTGCTTATATAGACCTC-3′). Thermocycling conditions were: 94°C for 2 minutes; 30 cycles of 94°C for 30 seconds, 55°C for 30 seconds, and 68°C for 4 minutes. A 1-μl aliquot of the second-round PCR product from NFL amplification (reservoir) or SGA amplification (rebound) was used as a template for env to which we aded CMV overhanging regions using the forward primer ENVfwd (5′-CACCGGCTTAGGCATCTCCTATGGCAGGAAGAA-3′) and the reverse primer envB3in or ENV N, depending on the subtype. The CMV promoter amplicon was fused to individual env genes via overlapping PCR with 10 ng of env and 0.5 ng of CMV using CMVenv primer and envB3in or ENV N as the reverse primer. Thermocycling conditions were: 94°C for 2 minutes; 20 cycles of 94°C for 30 seconds, 55°C for 30 seconds, 68°C for 4 minutes; followed by a final extension at 68°C for 10 minutes. All PCR reactions were carried out using Platinum Taq HiFi polymerase. Resulting amplicons were analyzed by gel electrophoresis, purified using the Macherey-Nagel gel and PCR purification kit, and co-transfected with the pSG3Δenv vector (NIH AIDS Reagent Program) into 293T cells to produce pseudoviruses as previously described^70^.

### Neutralization assays

Viruses were tested against broadly neutralizing antibodies (bNAbs) and purified autologous IgGs using the TZM-bl cell neutralization assay, as previously described^39,71^. All neutralization assays were conducted in laboratories adhering to Good Clinical Laboratory Practice (GCLP) quality assurance standards. Pseudovirus clones derived from both the reservoir and rebound phases were tested. For autologous antibody testing, baseline IgG was purified using Protein G Sepharose 4 Fast Flow (GE Life Sciences). Neutralization assays were performed using a starting maximum concentration of 50 μg/ml for 3BNC117 and 10–1074, and 500 μg/ml for autologous IgG, followed by eight five-fold serial dilutions. All experiments were conducted in duplicate.

## Extended Data

## Supplementary Material

Supplement 1 **Supplementary Table 1.** Quantification of HIV reservoir size by digital droplet PCR (ddPCR) and quantitative quadruplex four-probe PCR (Q4PCR) for individual participants.
**Supplementary Table 2.** Near full-length intact proviral sequences from participants in Arms A and B.
**Supplementary Table 3.** Neutralization sensitivities of selected proviruses in pseudovirus neutralization assays against baseline autologous IgG, 3BNC117, and 10–1074 from participants in both study arms.
**Supplementary Table 4.** Individual ENV coding region sequences obtained from plasma of participants in both study arms during rebounding viremia by single-genome amplification (SGA).
**Supplementary Table 5.** Neutralization sensitivities of selected rebound-derived pseudoviruses against baseline autologous IgG, 3BNC117, and 10–1074 from participants in both study arms.
**Supplementary Table 6.** Geometric mean neutralization sensitivities of reservoir-derived and rebound pseudoviruses from individual participants in both study arms, tested against baseline autologous IgG, 3BNC117, and 10–1074.
